# Axial resolution and imaging contrast enhancement in inverted light-sheet microscopy by natural illumination modulation

**DOI:** 10.3389/fnins.2022.1032195

**Published:** 2022-10-18

**Authors:** Zhi Wang, Wei Qiao, Tao Jiang, Siqi Chen, Bolin Lu, Kefu Ning, Rui Jin, Hui Gong, Jing Yuan

**Affiliations:** ^1^Wuhan National Laboratory for Optoelectronics, Britton Chance Center for Biomedical Photonics, Huazhong University of Science and Technology, Wuhan, China; ^2^MoE Key Laboratory for Biomedical Photonics, School of Engineering Sciences, Huazhong University of Science and Technology, Wuhan, China; ^3^HUST-Suzhou Institute for Brainsmatics, Jiangsu Industrial Technology Research Institute (JITRI) Institute for Brainsmatics, Jiangsu, China

**Keywords:** light-sheet microscopy, large-volume imaging, optical sectioning microscopy, natural illumination modulation, axial resolution enhancement, imaging contrast improvement

## Abstract

Inverted light-sheet microscopy (ILSM) is widely employed for fast large-volume imaging of biological tissue. However, the scattering especially in an uncleared sample, and the divergent propagation of the illumination beam lead to a trade-off between axial resolution and imaging depth. Herein, we propose naturally modulated ILSM (NM-ILSM) as a technique to improve axial resolution while simultaneously maintaining the wide field-of-view (FOV), and enhancing imaging contrast *via* background suppression. Theoretical derivations, simulations, and experimental imaging demonstrate 15% axial resolution increases, and fivefold greater image contrast compared with conventional ILSM. Therefore, NM-ILSM allows convenient imaging quality improvement for uncleared tissue and could extend the biological application scope of ILSM.

## Introduction

Inverted light-sheet microscopy (ILSM) utilizes axial parallel detection (Wu et al., 2011; Albert-Smet et al., 2019), instead of layer-by-layer imaging in the axial direction (Huisken et al., 2004), to accelerate the imaging of three-dimensional (3D) volumes. Nonetheless, the divergent propagation behavior of the illumination beam and strong scattering in biological tissue can deteriorate axial resolution and contrast at depths (Glaser et al., 2017), leading to quality degradation and depth limitation for volume imaging. Therefore, improving the axial resolution and imaging contrast of ILSM is crucial for large-scale 3D imaging without sacrificing imaging depth.

To address this problem, many techniques have been combined with ILSM. In single-photon-excited ILSM, strong tissue scattering broadens the thickness of the light sheet, which is the main factor affecting image quality. Optical clearing effectively reduces tissue scattering by homogenizing the refractive indices of the tissue components. Nevertheless, clearing treatments often take hours or days, do not always eliminate tissue scattering, and can result in expansion or shrinkage, leading to inevitable morphological distortion (Ueda et al., 2020).

Synchronous beam scanning and rolling-shutter detection have been introduced to ILSM to suppress the defocused background (Wang et al., 2019). However, accurate scanning requires complex synchronization of digital communication (Kim et al., 2021). Multi-view light-sheet microscopy provides another approach for overcoming image resolution inhomogeneity. However, this method is difficult to achieve owing to the requirement for high-accuracy detection switching and complex image fusion (Wu et al., 2016). Structured illumination has also been combined with ILSM to improve image quality (Wang et al., 2021). In this case, an extra modulator increases the complexity of the system, and the acquisition of multiple raw images is necessary, which results in reduced effective imaging throughput. Subtraction has been employed in several kinds of microscopies to enhance high-frequency signals and suppress low-frequency signals, and then improve the spatial resolution and imaging contrast, due to its simplicity in image reconstruction and system configuration compared with the above methods (Heintzmann et al., 2003; Wang and Kobayashi, 2014). However, previous subtraction used in light-sheet microscopy still needs an extra modulator and switchable illumination in the system and then increases system complexity (Jia et al., 2019; Deng et al., 2021). Natural illumination modulation firstly proposed by line illumination modulation (LiMo) microscopy simplifies the modulation (Zhong et al., 2021).

Here, we propose a technique called naturally modulated ILSM (NM-ILSM) designed to improve both axial resolution and imaging contrast in the 3D imaging of uncleared tissue. NM-ILSM employs natural illumination modulation along the propagation direction of the Gaussian light-sheet beam to image the sample using different point spread functions (PSFs) and reconstructs the image by subtraction. This method employs the same configuration as conventional ILSM—no additional components are required, and hence measurements are easily realized. We theoretically analyzed and determined the PSF to demonstrate the resolution and contrast enhancements afforded by NM-ILSM. We experimentally verified our analysis by imaging fluorescent beads. We then compared the optical sectioning capabilities of NM-ILSM with ILSM and deconvolution methods by imaging mouse brain slices and blocks. All the results indicate that our method improves axial resolution and imaging contrast, broadens the scope of ILSM imaging, and facilitates applications in 3D biological visualization.

## Materials and methods

### Sample preparation

A total of 3–6 months-old heterozygous Thy1-GFP-m mice (Jackson Laboratory) with a significant presence of expressing green fluorescent protein (GFP) in the neurons were used in the study. The mice were kept in a 12-h dark/light cycle with food and water provided *ad libitum*.

The mice were anesthetized with a 1% solution of sodium pentobarbital *via* intraperitoneal injection and intracardially perfused with 0.01 M phosphate-buffered saline (PBS, Sigma-Aldrich), which was followed by 4% paraformaldehyde (PFA, Sigma-Aldrich) in 0.01 M PBS. Then, the brains were excised and post-fixed in 4% PFA at 4°C for 24 h. After fixation, each intact brain was rinsed overnight at 4°C in 0.01 M PBS and prepared for embedding.

Oxidized agarose was made according to the following steps. Agarose type I-B (Sigma-Aldrich) was added to 10 mM sodium periodate (NaIO4, Sigma-Aldrich) solution and stirred for 2–3 h at room temperature. The oxidized product was repeatedly washed and resuspended in PBS to bring the final concentration to 5%. The brains were pat-dried and embedded in melted oxidized agarose using a silicone mold. There were several cuboid-shaped grooves in the mold for brain embedding and gridlines for correcting the brain orientation. The mold and brain were placed in a 55°C water bath for 0.5 h until the surfaces of the brain were fully coated with agarose. During the water bath, the orientation of the brain could be easily adjusted. Then, the mold and brain were left at room temperature for 0.5 h to allow the agarose to solidify. After that, the brain was separated from the mold and stored in PBS at 4°C before imaging.

### Optical setup

**FIGURE 1:**
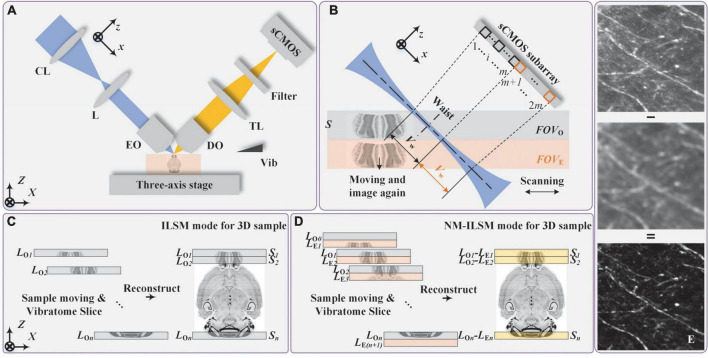
Imaging principle of NM-ILSM. **(A)** Schematic diagram of the system configuration. **(B)** ILSM mode: imaging the sample *S* with *FOV*_O_. NM-ILSM mode: imaging the sample *S*_*n*_ again after ILSM mode with *FOV*_E_. CL, cylindrical lens; L, lens; EO, excitation objective; DO, detection objective; TL, tube lens; sCMOS, scientific complementary metal–oxide–semiconductor detector. **(C,D)** Data acquisition flow of large-volume 3D imaging by the updated system combined with a customized vibratome in ILSM and NM-ILSM modes, respectively. **(E)** A typical example showing maximum intensity projections (MIP) with a 20-μm-thickness in the central and extended FOVs, and their NM-ILSM reconstructed image.

The imaging system, shown schematically in Figure 1A, was designed to facilitate measurements *via* two modes of operation: ILSM and NM-ILSM. A light source (not shown in Figure 1A) was used to provide an expanded ellipse beam with a semi-major and a semi-minor axis of 10 and 20 mm, respectively. A cylindrical lens (CL; LJ1267RM-A, Thorlabs) compressed the beam into a light sheet. After a doublet lens (L; AC254-200-A, Thorlabs) and excitation objective (EO; UMPLFLN10XW, Olympus), the light sheet was focused on the sample surface at an oblique angle of 45°. The excited fluorescence signal was collected by a detection objective (DO; UMPLFLN10XW, Olympus) aligned perpendicular to the illumination direction, transmitted through a tube lens (TL; TTL180-A, Thorlabs), and a single-band bandpass filter (FF01-520/35-25, Semrock), and then detected by a scientific complementary metal–oxide–semiconductor (sCMOS) camera (ORCA-Flash4.0, Hamamatsu) operated in subarray mode as a multiline detector. The field-of-view (FOV) in ILSM mode *FOV*_*O*_ contains *m* lines of pixels around the waist of the illumination beam. NM-ILSM extended the FOV along the beam propagation direction to the other *m* lines, called *FOV*_*E*_. The sample is imaged twice in *FOV*_*O*_ and *FOV*_*E*_ in NM-ILSM mode. A three-axis stage (*X*-axis: XML210, *Y*-axis: XMS100, *Z*-axis: GTS30V, Newport) was used to accurately control the sample movement in three dimensions.

### Natural modulation by Gaussian beam

Naturally modulated ILSM utilizes the spatial intensity distribution difference of the illumination beam along the propagation direction to achieve the natural modulation. In theory, we can effectively remove the background by imaging the sample surface twice in the *FOV*_*O*_ and *FOV*_*E*_ and then subtracting the two images (in Figure 1B).

As the beam diameter varied along the *x* illumination direction, the *i*-th line (1 ≤ *i* ≤ 2*m*) of the detector recorded the *i*-th layer of the sample with *PSF*_*i*_, a PSF varying with *i*. Images captured by the *i*-th line of the detector, *I*_*i*_(*S*), can be expressed as:


(1) $$I_{i}\left(S\right)=S⊗PSF_{i}+N_{0}\left(1\leq i\leq 2m\right),$$

where ⊗ represents convolution; *S* is the excited fluorescence signal at the focus and *N*_0_ is the background noise caused by the scattering in the sample or imperfections in the optics (Warren et al., 2013). In conventional ILSM, the waist area of the illumination beam is used to image sample *S* with *FOV*_*O*,_ and *S* is recorded as a 3D image *L*_*O*_.

To achieve illumination modulation in NM-ILSM, *S* is moved to the adjacent extended FOV, *FOV*_*E*_. And the additional pixels of the detector record *S* as *L*_*E*_, a 3D image with another modulation. The *FOV*_*O*_ width *V*_*w*_, which is mapped onto *m* lines of the detector, determines the distance by which the sample is moved between these two acquisitions. The PSFs at the *j*-th line of *FOV*_*O*_ and *FOV*_*E*_ satisfy:


(2) $$\left\{ \begin{array}{c} P\,S\,F_{\text{O}}\left(j\right)=P\,S\,F_{j}\\ P\,S\,F_{\text{E}}\,\left(j\right)=P\,S\,F_{j+m} \end{array}\right.$$

The defocus noise is removed by subtraction, by obtaining the difference between these two images of the same sample. NM-ILSM effectively improves the signal-to-background ratio (SBR). However, the shot noise introduced by the background signal remains in the result. Therefore, the change in SNR is limited. The optically sectioned NM-ILSM image $I_{j}^{\text{NM}-\text{ILSM}}$ of *S* is expressed as:


(3) $${\text{I}}_{j}^{N\,M-I\,L\,S\,M}\,\left(S\right)=\text{S}⊗\left(\text{P}\,S\,F_{j}-\text{P}\,S\,F_{j+m}\right)$$

Therefore, the equivalent PSF of NM-ILSM (*PSF*_*N*_) is acquired by substituting Eq. 2 into Eq. 3:


(4) $$P\,S\,F_{\text{N}}\,\left(j\right)=P\,S\,F_{\text{O}}\,\left(j\right)-P\,S\,F_{\text{E}}\,\left(j\right)=P\,S\,F_{j}-P\,S\,F_{j+m}$$

The enhancement of axial resolution and imaging contrast are theoretically derivated in Supplementary Sections 1, 2.

### Image acquisition method for large sample

Naturally modulated ILSM can be combined with a vibratome (Jiang et al., 2017) to further extend the imaging depth, as is also the case for conventional ILSM (Yang et al., 2018). The data acquisition flow for imaging a large sample in ILSM mode is shown in Figure 1C. In the first scanning, the first thick layer *S*_1_ on the sample surface is imaged to be *L*_*O*_*_1_* in *FOV*_*O*_, then the sample is lifted and the next layer *S*_2_ is exposed and imaged as *L*_*O*_*_2_*. Optical imaging and mechanical slicing are performed alternately until data acquisition is complete. In NM-ILSM mode shown in Figure 1D, we simultaneously record two images *L*_*O*_*_*n*_* and *L*_*E*_*_(n+1)_* of adjacent layers *S*_*n*_ and *S*_*n* + 1_, respectively, with the extended subarray of the detector. Then, the sample is lifted by one layer and the layer *S*_*n* + 1_ is imaged with another illumination part in *FOV*_*O*_ and recorded as *L*_*O*(_*_*n*_*_+ 1)_. So this approach only requires one extra scanning compared with ILSM, and two entire datasets generated by different PSFs are obtained. We process the NM-ILSM data by subtraction to generate each single-plane image. The reconstructed FOV of NM-ILSM is the same as the *FOV*_*O*_ of ILSM. We then concatenate the individual images of single planes to reconstruct the 3D image dataset of a large sample, as shown in Figure 1E.

## Results

### System characteristics simulation

**FIGURE 2:**
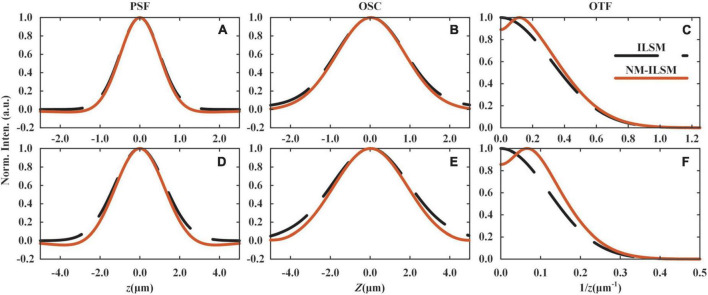
Simulated imaging performances of ILSM and NM-ILSM. Axial PSFs, OSCs, and OTFs at the center **(A–C)** and the edge **(D–F)** of the FOV.

We evaluate the system PSF, optical sectioning capacity (OSC), and illumination optical transfer function (OTF), as shown in Figure 2. The detailed derivation is described in Supplementary Sections 1, 2. To satisfy a high throughput requirement for large 3D sample imaging, NM-ILSM generated a light sheet with a waist radius of 1.0 μm and chose the line number *m* to be 24 for acquiring as large a FOV as possible in all the following experiments. The corresponding *V*_*w*_ and imaging depth in the *Z* direction were 31.2 and 22.1 μm, respectively. In this case, the sample in *FOV*_*E*_ is illuminated by a thicker light sheet, and the axial detection range is mainly limited by the depth of focus of the DO. The sample structures detected with *FOV*_*E*_ are not significantly different from *FOV*_*O*_, while the intensity is much smaller. Therefore, the subtraction method can effectively remove the background without causing the loss of structural information. In addition, to increase the signal intensity, we operated the camera with 2 × 2 binning modes. Thus, the voxel size in Cartesian coordinates was 1.3 μm × 1.3 μm × 0.9 μm. The camera works at the maximum transmission throughput of 1,936 fps. The sectioning speed was 0.5 mm/s.

Figures 2A,B show the PSFs and OSCs of ILSM and NM-ILSM at the center of the FOV. The shrinking of the curves indicates a better axial resolution of NM-ILSM. The negative values in the curves are inevitable and set to be zero, like most subtraction schemes (Heintzmann et al., 2003; Li et al., 2013; Segawa et al., 2014). According to the references (Ge et al., 2015; Yoshida et al., 2019), this process causes little distortion when the PSF is above −0.2. In our case, the PSF valleys are far above −0.2, allowing the process acceptable. We further calculated the OTFs of the illumination arm in ILSM and NM-ILSM, as shown in Figure 2C. Although NM-ILSM did not extend the cut-off frequency, it reshaped the OTF by low-frequency rejection and high-frequency enhancement, leading to resolution enhancement. Similarly, NM-ILSM reshaped the PSF, OSC, and OTF at the edge of the FOV as shown in Figures 2D–F, indicating the resolution enhancement works throughout the whole FOV.

### Simulation of spoke-like sample imaging

**FIGURE 3:**
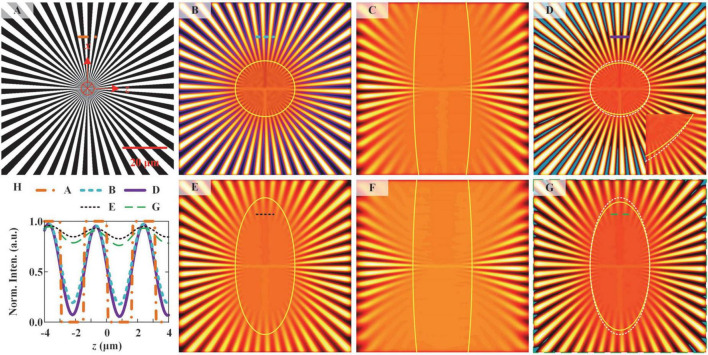
Simulated imaging of a spoke-like sample by ILSM and NM-ILSM. **(A)** Spoke-like sample. ILSM image was acquired at the beam waist **(B)** and 20 μm from the beam waist **(C)**. **(D)** NM-ILSM image was acquired at the beam waist. ILSM images were acquired at distances of 10 μm **(E)** and 30 μm **(F)** from the beam waist. **(G)** NM-ILSM image was acquired at a distance of 10 μm from the beam waist. **(H)** Normalized intensity profiles along the correspondingly colored lines in **(A–G)**.

We simulated the ILSM and NM-ILSM imaging of a spoke-like sample with the formulas described in Supplementary Section 1 and shows the results in Figure 3. Spoke-like sample (Figure 3A) is widely used to assess imaging quality (Mudry et al., 2012; Kuang et al., 2013). NM-ILSM offers an improvement only in the *z*-direction resolution. We simulated ILSM and NM-ILSM images at *xz* plane by an 80 μm × 80 μm spoke-like sample, as shown in Figures 3B–G. To show the resolution, we drew the boundary of the unresolved areas with the Rayleigh criterion (Born and Wolf, 1980) by the yellow boundaries in Figures 3B–G. Out of the boundary, the valley-to-peak ratio between the two lines is less than 73.5%. Figures 3B,C show the simulated ILSM images at the centers of *FOV*_*O*_ and *FOV*_*E*_, respectively. Figure 3D shows the NM-ILSM image reconstructed from Figures 3B,C. Figure 3D also shows the boundary of the unresolved area in Figure 3B as a white dashed circle to demonstrate the imaging resolutions of ILSM. The unresolved area is smaller by 5.6% with NM-ILSM at the center of FOV, which is outlined by the yellow circle in Figure 3D.

The resolution improvement of NM-ILSM applies to the entire FOV. We simulated the ILSM images generated at the edges of *FOV*_*O*_ and *FOV*_*E*_, as shown in Figures 3E,F. We then reconstructed the corresponding NM-ILSM image, as shown in Figure 3G. The result demonstrated a 7.9% shrinking of the unresolved region by NM-ILSM at the edge of FOV. All the results demonstrate the axial resolution improvement by the use of NM-ILSM.

Figure 3H shows the normalized intensity profiles along the correspondingly colored lines at the same positions. We also calculated the image contrast from these correspondingly colored lines as follows:


(5) $$\text{C}\,o\,n\,t=\frac{{\text{I}}_{m\,a\,x}-{\text{I}}_{m\,i\,n}}{{\text{I}}_{m\,i\,n}},$$

where *I*_*min*_ and *I*_*max*_ are the minimum and maximum intensities, respectively, along the lines. The contrast was increased from 5.1 to 24.7 for the image at *x* = 0 μm and from 0.2 to 0.4 at *x* = 10 μm, as the curves shown in Figure 3H.

Negative values are also inevitable in the simulation, indicated by blue areas in Figures 3D,G. They were always located in the background areas, indicating there is no signal in the negative areas. The number of spokes also remained unchanged in NM-ILSM. These phenomena demonstrated that the negative intensities in the PSF cause little distortion and were allowed to be set to zeros (Dehez et al., 2013).

### System performance demonstration with model sample

**FIGURE 4:**
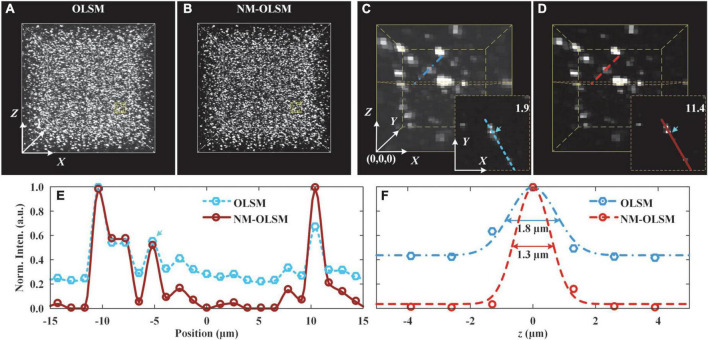
Images of agarose-embedded 200-nm-diameter fluorescent beads. **(A,B)** Visualizations of 0.3 mm × 0.3 mm × 0.3 mm image stacks were captured by ILSM and NM-ILSM, respectively. **(C,D)** Expanded views of 30 × 30 × 40-pixel subblock of the stacks in **(A,B)**, respectively. The subimages at the lower right corners show the planes at Z = 20.2 μm of the subblocks. **(E)** Normalized intensity distribution across several beads outlined in **(C,D)**. **(F)** Normalized intensity distribution along the *z*-axis of the typical bead.

To experimentally compare the imaging contrasts and resolutions of NM-ILSM and ILSM, we imaged 0.2-μm-diameter yellow-green fluorescent beads (F8811, Thermofisher) using these two imaging modalities (Figure 4).

Before imaging, we dispersed 80 μl of a solution of the beads in 10 ml of heated 4% agarose solution. After the solution solidified, we obtained an agarose-embedded sample with randomly sparsely distributed beads. Using the imaging parameters detailed in section “Results,” we acquired two 0.3 mm × 0.3 mm × 0.3 mm blocks, using ILSM (Figure 4A) and NM-ILSM (Figure 4B), respectively.

We quantized the imaging contrast enhancement of NM-ILSM for intense structures. The 30 × 30 × 40-pixel blocks shown in Figures 4C,D contain several beads very close together. We show the planes of *Z* = 20.2 μm of the data blocks at the lower right corners and extract the intensity along the line to show the signals in Figure 4E. To evaluate the system performance, we calculated the contrast with the intensities around the signal shown in the box of Figure 4E. ILSM blurred the weak bead with a low contrast of 1.9, and NM-ILSM improved the contrast to 11.4.

The intensities of a randomly selected bead on the lines in Figures 4C,D are shown in Figure 4F. NM-ILSM shrank the full width at half maximum (FWHM) after Gaussian fitting from 1.8 μm of ILSM to 1.3 μm, demonstrating the PSF improvement.

### Imaging contrast improved with sliced tissue

**FIGURE 5:**
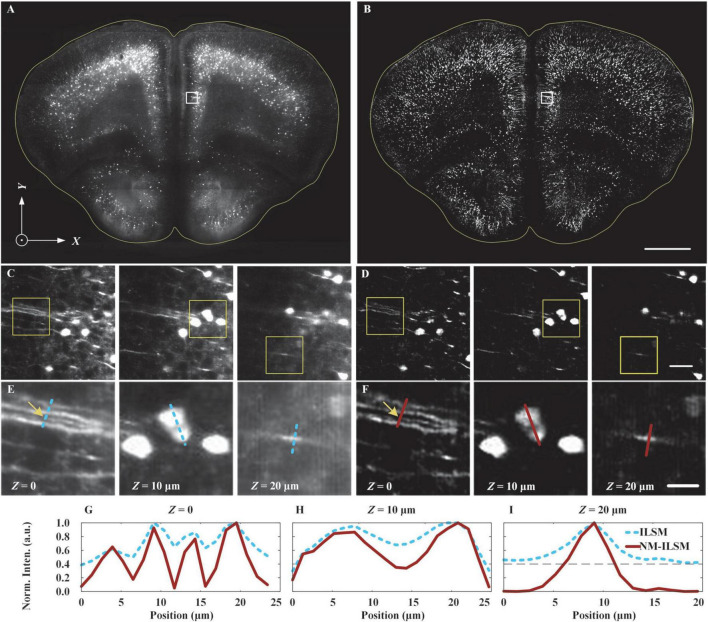
Inverted light-sheet microscopy and NM-ILSM image quality comparison. ILSM **(A)** and NM-ILSM **(B)** MIP images for a 20-μm-thick coronal slice of a Thy1-GFP mouse brain. Scale bar: 1 mm. **(C,D)** Expanded views at depths of *Z* = 0, 10, and 20 μm (left to right) of the areas outlined by white boxes in **(A,B)**, respectively. Scale bar: 50 μm. **(E,F)** Expanded views of the areas outlined by the yellow boxes in **(C,D)**, respectively. Scale bar: 20 μm. **(G–I)** Normalized intensity profiles along the correspondingly colored lines in **(E,F)**.

To evaluate the improvement offered by NM-ILSM in visualizing uncleared biological tissue slices, we imaged a 20-μm-thick agarose-embedded slice of brain tissue from a Thy1-GFP mouse (007919, Jackson Laboratory), as shown in Figure 5.

Figures 5A,B show maximum intensity projection (MIP) ILSM and NM-ILSM images of the sample, respectively. In comparison, the NM-ILSM image (Figure 5B) has a suppressed background. Expanded views of the areas outlined by the white boxes in Figures 5A,B, at the sample surface as well as 10 and 20 μm below the surface, are shown in Figures 5C,D, respectively, and these further verify the greater background suppression and imaging contrast of NM-ILSM compared with ILSM. Further view expansions, corresponding to the areas outlined by the yellow boxes in Figures 5C,D, are shown in Figures 5E,F, respectively. In addition, in Figures 5G–I, we show the normalized intensity profiles along the colored lines overlaid through the dendrites and somas in Figures 5E,F. The signals are more distinct and sharper in the NM-ILSM images, which benefit from better suppression of the defocused background. The fibers marked by the blue line in Figure 5E had a low contrast of 1.6, while the contrast is improved to be 19.0 in NM-ILSM, as the intensities shown in Figure 5G. NM-ILSM also improves the contrast of the cells from 0.4 of ILSM to 1.5, as the intensities are shown in Figure 5H. The normalized background is suppressed from around 0.4 to be close to zeros, as shown in Figure 5I.

### Observation of large tissue block with faster image improvement than deconvolution

**FIGURE 6:**
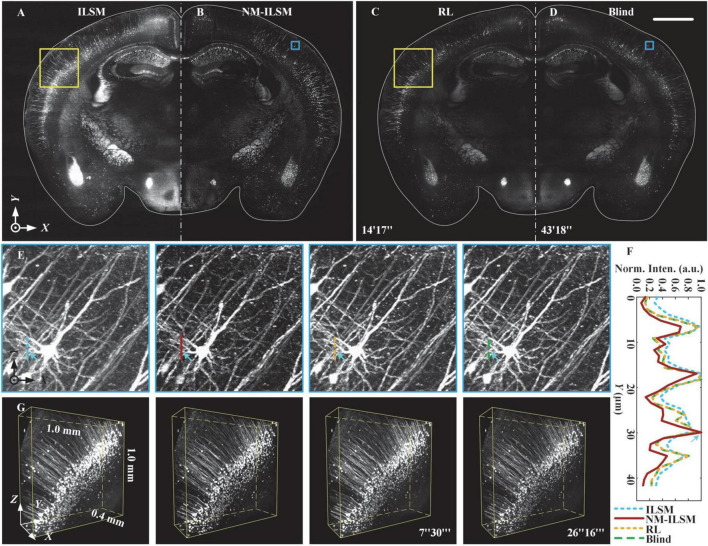
Inverted light-sheet microscopy, NM-ILSM, RL deconvolution, and blind deconvolution 3D imaging quality comparison for thick brain tissue. **(A–D)** Normalized MIPs of a 20-μm-thick coronal (*XY*) plane. Scale bar: 1 mm. **(E)** Normalized MIPs of a 200 × 200 × 144-pixel block in the blue boxes in **(A–D)**. **(F)** Normalized intensity profiles along the colored lines in **(E)**. **(G)** Three-dimensional visualization of volumes in the cortex indicated by the white boxes in **(A–D)**.

To evaluate the feasibility of NM-ILSM for 3D structure visualization of uncleared thick biological tissue, we imaged a 12.0 mm × 8.2 mm × 0.4 mm agarose-embedded block of Thy1-GFP mouse brain tissue (007919, Jackson Laboratory) by ILSM and NM-ILSM, as shown in Figure 6.

Naturally modulated ILSM acquired the block by 21 layers of scanning, one layer more than ILSM. The acquisition included imaging, slicing, and moving the sample, and was performed in 30.3 min. To verify the imaging quality improvement of NM-ILSM, we show 20-μm-thick MIPs of the tissue acquired using ILSM, NM-ILSM, RL deconvolution, and blind deconvolution in Figures 6A–D, respectively. The computing platform for deconvolutions was configured with an Intel^®^ Xeon^®^ E5-2587W v2 CPU at 3.40 GHz with 256 GB of memory. As Figures 6A–D indicated, ILSM had a strong background, which was suppressed by NM-ILSM and deconvolutions. The RL and blind deconvolutions took 14 and 43 min for the single layer, respectively. Those processing times were much longer than the extra acquiring time of NM-ILSM. We sequentially zoomed in to show the 130-μm-thick MIPs containing a neuron in Figure 6E. The intensities of the fibers under the lines are shown in Figure 6F. The fiber indicated by the arrows was drowned in the background in ILSM, while NM-ILSM brought it out with a smaller FWHM. The image quality improvement was similar to the deconvolutions. We further show the normalized 1.0 mm × 1.0 mm × 0.4 mm subblocks in Figure 6G outlined by the white boxes in Figures 6A–D. The biological structures remained continuous in these images. Somas and dendrites were more distinct with NM-ILSM and the deconvolutions than those with ILSM, while NM-ILSM took much less time to process the 3D image. The results suggest that NM-ILSM combined with tissue sectioning provides a new, fast, quality-improved approach for 3D high-contrast visualization of uncleared biological tissue.

## Discussion

In this report, we proposed a simple method to improve the axial resolution and imaging contrast of ILSM using the natural modulation based on the intensity variation along the light-sheet propagation direction. Our theoretical derivation, simulated imaging of a spoke-like sample, and experimental imaging of fluorescent beads and biological tissue all demonstrated that NM-ILSM has an enhanced high-frequency response and greater background suppression compared to ILSM. The simulation and experiment verified the resolution and contrast enhancement of NM-ILSM, and the tissue structure remained continuous.

To our knowledge, this study is the first trial of applying natural modulation to light-sheet microscopy. It takes advantage of the axial parallel detection of ILSM and achieves high-throughput 3D imaging of large-scale samples combined with tissue vibratome sectioning. In addition, NM-ILSM is easily achieved without the need for any extra modulators or burden calculation and achieves a similar effect of high-frequencies-enhancing with deconvolutions. While, our method is much simpler and faster due to avoiding the heavy and time-consuming iteration, especially for large 3D samples.

Naturally modulated ILSM is valid for the hypothesis that the Gaussian-profile illumination is scattered to be uniform at defocused positions in both *FOV*_O_ and *FOV*_E_. In the case that the two views are too close to each other, subtracting them may cause a great loss of signal. In the other case of two large FOVs, the light sheet in *FOV*_E_ is much thicker but weaker than the one in *FOV*_O_. Since imaging is also limited by the depth of focus in the detection path, we do not lose much structural information due to subtraction. We experimentally chose a proper parameter of the width of FOV to satisfy the above hypothesis. Furthermore, the scattering and absorption may also introduce a large difference in the observed images of two FOVs at large depths compared to the light-sheet Rayleigh range. Therefore, we combined tissue mechanical sectioning to keep imaging at the sample surface for a 3D large sample to avoid scattering and absorption from deep tissue.

In addition, for imaging the sample in a single layer, NM-ILSM may have the potential effect of photobleaching and speed loss on sensitive samples due to twice-imaging, like wide-field structured illumination microscopy. While, for 3D imaging, our method allows us to simultaneously detect the signals from adjacent layers using the different parts of the illumination. If the fluorescence labels are bright enough to support the ultimate-speed detection, NM-ILSM needs double imaging time compared with ILSM due to doubling the subarray width and halving the frame rate. In the case of imaging weak signals, NM-ILSM works with the same exposure time and frame rate as ILSM and spends almost the same whole data acquisition time without extra photobleaching or speed loss.

In conclusion, NM-ILSM potentially provides the capacity to visualize large uncleared 3D biological tissue samples with improved imaging quality.

## Data availability statement

The raw data supporting the conclusions of this article will be made available by the authors, without undue reservation.

## Ethics statement

The animal study was reviewed and approved by the Institutional Animal Ethics Committee of Huazhong University of Science and Technology.

## Author contributions

JY conceived of and organized the research. ZW performed data acquisitions of all the measurements. JY, WQ, and RJ discussed the theory of the imaging enhancement method. TJ and HG provided the vibratome. SC provided the biological sample. BL and KN performed the image process and evaluation. JY, WQ, RJ, and ZW drafted the manuscript. All authors reviewed and approved the final manuscript.
