# Supplementary material for: Axial resolution and imaging contrast enhancement in inverted light-sheet microscopy by natural illumination modulation
Source: Front Neurosci. 2022 Oct 18;16:1032195. doi: 10.3389/fnins.2022.1032195 (PMC9623180; doi:10.3389/fnins.2022.1032195)
Supplement: Supplementary file 1 [file Data_Sheet_1.pdf]

Supplementary Material

Section 1. Axial resolution enhancement

To evaluate the axial resolution enhancement provided by NM-ISLM (**Figures 2,3**), we analyzed and compared the PSFs of NM-ISLM and OLSM in the *z* direction. In general, the point spread function (PSF) of an imaging system, is determined by both its illumination PSF (PSF_{ill}) and its detection PSF (PSF_{det}):

$$\boldsymbol{PSF = PSF_{ill} \times PSF_{det}}, \tag{S1}$$

where PSF_{ill} represents the intensity distribution of the illumination beam, and PSF_{det} is decided by the objective and detector pixels, i.e., a 3D constant. We only consider the axial difference between the illuminations of the two methods to simplify the derivation here. Taking scattering and absorption into consideration (Petrova and Petrov, 2018), we compared the normalized PSF_{ill} for these two methods:

$$\begin{cases} PSF_{\text{ill}}^{\text{ISLM}} = exp\left(-\frac{2c_z^2}{\omega_1^2}\right) \\ PSF_{\text{ill}}^{\text{NM-ISLM}} = \frac{\omega_2^2 exp\left(-\frac{2c_z^2}{\omega_1^2}\right) - \alpha_t \omega_1^2 exp\left(-\frac{2c_z^2}{\omega_2^2}\right)}{\omega_2^2 - \alpha_t \omega_1^2} \end{cases}, \tag{S2}$$

where c_z is the axial position. The factor α_t ($\alpha_t < 1$) is the intensity reduction ratio relative to the ideal condition without absorption and scattering. The radii of the Gaussian beam at arbitrarily selected positions in the FOV_{O} and FOV_{E} are ω_1 and ω_2, respectively. They satisfy:

$$\begin{cases} \omega_1 = \omega_0 \sqrt{1 + \left(\frac{x_1}{R}\right)^2} \\ \omega_2 = \omega_0 \sqrt{1 + \left(\frac{x_2}{R}\right)^2}, \\ R = \frac{\pi \omega_0^2}{\lambda_e} \end{cases} \tag{S3}$$

where ω_0 is the beam waist, x_1, x_2 are the distance of the positions from the waist, *R* is the Rayleigh range of the beam, and λ_e is the excitation wavelength. As $|x_2| > |x_1|$, we simplify the relationship of the radii as:

$$\boldsymbol{\omega_1 = k\omega_2}. \tag{S4}$$

where the factor *k* always satisfies $0 < k < 1$.

To further analyze the distributions of PSF_{ill} for both methods, we calculated the optical transfer function (OTF, the Fourier transform of the PSF) along the *z* direction. As OTF is limited by the cutoff frequency of the incoherent imaging system, we simplify the analysis by the Taylor series. When the spatial frequency in the *z* direction (w_z) goes to zero, OTFs in the illumination arm of ISLM, $OTF_{\text{ill}}^{\text{ISLM}}$, and NM-ISLM, $OTF_{\text{ill}}^{\text{NM-ISLM}}$ respectively, are expressed as:

$$\begin{cases} \boldsymbol{OTF_{\text{ill}}^{\text{ISLM}}} = \sqrt{\frac{\pi}{2}}\boldsymbol{\omega_1(1-\frac{\omega_1^2}{8}w_z^2)} \\ \boldsymbol{OTF_{\text{ill}}^{\text{NM-ISLM}}} = \sqrt{\frac{\pi}{2}}\frac{\omega_1\omega_2}{\omega_2^2-\alpha_t\omega_1^2}\boldsymbol{[\omega_2-\alpha_t\omega_1+\frac{\omega_1\omega_2}{8}\alpha_t w_z^2(\alpha_t\omega_2-\omega_1)]} \end{cases}. \tag{S5}$$

As Eq. (S5) shows, if $\alpha_t\omega_2$-ω_1 > 0 ($\alpha_t > k$), $OTF_{\text{ill}}^{\text{NM-ISLM}}$ increases when $w_z \approx 0$. The changing trend indicates that the low-frequency response of NM-ISLM is suppressed compared with $OTF_{\text{ill}}^{\text{ISLM}}$. If $\alpha_t\omega_2$-$\omega_1 \leq 0$ ($\alpha_t \leq k$), these two OTFs both peak at $w_z = 0$. The trends of the normalized OTFs are shown by the derivations, and satisfy:

$$\frac{\frac{dOTF_{\text{ill}}^{\text{ISLM}}}{dw_z}}{\frac{dOTF_{\text{ill}}^{\text{NM-ISLM}}}{dw_z}} = \frac{-\frac{\omega_1^3}{4}w_z}{-\frac{\omega_1^2\omega_2^2(\omega_1-\alpha_t\omega_2)}{4(\omega_2^2-\alpha_t\omega_1^2)}w_z} = \boldsymbol{1} + \frac{\alpha_t(1-k^3)}{k-\alpha_t} > \boldsymbol{1}. \tag{S6}$$

As Eq. (S6) shows, NM-ISLM slowers the decrease of OTF compared to ISLM, when $|w_z|$ increases. It indicates that the high-frequency response is relatively enhanced of NM-ISLM, as shown in Fig. 1(d), i.e., NM-ISLM improves the axial resolution in comparison with ISLM.

Section 2. Imaging contrast enhancement

To evaluate the imaging contrast enhancement (**Figures 2**), we also derived and compared the optical sectioning capability (OSC) of ISLM and NM-ISLM. OSC is measured by the spectral response of an ideal fluorescent lamina with no thickness. As the intensity of the ideal light sheet is uniform in the *y* direction, we calculated the frequency response of the lamina (Poher et al., 2008):

$$\boldsymbol{I(u,\beta) = C\int_{-2}^{2} G(s,u)T(s,u)sinc(\frac{as}{2\pi})exp(-is\beta)ds}, \tag{S7}$$

where C is a constant, $G(s,u)$ is the Fourier transform of the illumination intensity, and s, u, a, and β are defined as follows:

$$\begin{cases} \boldsymbol{s} = \frac{\lambda}{nsin(\alpha)}\boldsymbol{f_x} \\ \boldsymbol{u} = \frac{8\pi n}{\lambda}\boldsymbol{c_z sin^2(\frac{\alpha}{2})} \\ \boldsymbol{a} = \frac{2\pi nsin(\alpha)}{\lambda}\boldsymbol{p} \\ \boldsymbol{\beta} = \frac{2\pi nsin(\alpha)}{\lambda}\boldsymbol{\Delta x} \end{cases}. \tag{S8}$$

In Eq. (S8), Δx is the lateral shift between illumination and detection, f_x is the lateral spatial frequency, λ is the fluorescent wavelength. p is the pixel size of the detector, and $n\sin(\alpha)$ is the numerical aperture (NA) of the objective. The Stokseth approximation $T(s,u)$ is used to estimate the defocus variation (Stokseth, 1969). According to the 3D intensity distribution of the Gaussian beam, the spectrum of illumination in the propagation direction will be concentrated in the region close to 0, so we approximate $G(s,u)$:

$$\mathbf{G}(s,u) \approx \begin{cases} exp(-bu^2), (s < s_1) \\ 0, (s > s_1) \end{cases}, \tag{S9}$$

where b is an artificially determined constant through the simulation which is related to the beam waist, and s_1 is a small constant. Based on Eq. (S9), we simplify and integrate Eq. (S7) from a small constant u_1 to infinity to estimate the impact of the whole defocus background:

$$\begin{cases} \int_{u_1}^{\infty} I^{\mathrm{ISLM}}(u)du \propto \int_{u_1}^{\infty}\int_0^2 G(s,u)\frac{J_1(us)}{us}dsdu \\ \qquad \propto \int_{u_1}^{\infty} exp(-bu^2)\frac{1}{u}\int_0^{s_1 u}\frac{J_1(t)}{t}dtdu = O(u_1^{-2}exp(bu_1^2)) \\ \int_{u_1}^{\infty} I^{\mathrm{NM-ISLM}}(u)du \propto \int_{u_1}^{\infty}\int_0^2 G(s,u)\frac{J_1(us)}{us}s^2dsdu \\ \qquad \propto \int_{u_1}^{\infty} exp(-bu^2)\frac{1}{u^3}\int_0^{s_1 u} J_1(t)t\,dtdu = O(u_1^{-5/2}exp(bu_1^2)) \end{cases}, \tag{S10}$$

where $J_1(t)$ is the first-kind, first-order Bessel function, and t is the simplification of us. Eq. (S10) shows that NM-ISLM introduces a higher-order factor than ISLM, leading to stronger background suppression and better contrast for NM-ISLM than for ISLM.

References

Petrova, T., and Petrov, Z. (2018). Modelling the Distribution of Lasers in Biological Tissues. *Int. J. Bioautomation* 22(3)**,** 213-228.

Poher, V., Kennedy, G.T., Manning, H.B., Owen, D.M., Zhang, H.X., Gu, E., et al. (2008). Improved sectioning in a slit scanning confocal microscope. *Opt. Lett.* 33(16)**,** 1813–1815.

Stokseth, P.A. (1969). Properties of a Defocused Optical System. *Journal of the Optical Society of America* 59(10)**,** 1314-1321. doi: 10.1364/JOSA.59.001314.
